# Educating the bystander: How contributing to ward rounds as a junior doctor or medical student can be helpful in preparation for clinical responsibilities Former l’observateur : de quelle manière la participation aux tournées des patients peut-elle aider le résident junior ou l’étudiant en médecine à se préparer aux responsabilités cliniques

**DOI:** 10.36834/cmej.70341

**Published:** 2020-09-23

**Authors:** Charlotte Sinclair, Chandra Biyani

**Affiliations:** 1St James’s University Hospital, Leeds, West Yorkshire, United Kingdom

During my first year as a qualified doctor (CS) after graduating medical school I had the opportunity to take part in the 4^th^ Urology Simulation Boot Camp, running from 8th October to 12th October 2018.

The urology boot camp is a 5-day course for doctors who are in their first year of surgical training for urology (mostly four years after medical school). During the course they take part in practical simulations, comprised of eight modules spanning technical and non-technical skills and covering the 1^st^ Year urology curriculum. The boot camp aims to aid the progression of urology trainees. However, as a very new junior doctor I felt the opportunity to be involved in a training program would benefit my own growth as a doctor.^1^^,^^2^

My role during this week was to facilitate the non-technical skills module which involved a simulated ward round, I would take the role of a first year junior doctor, whilst we saw a number of patients (actors) with urological problems led by the urology trainee doctor (delegate). We were accompanied by a nurse, health care assistant and another delegate acting as a second junior doctor. During the ward round the delegates had to deal with a number of realistic interruptions which included bleeps from various other wards requesting help; patients becoming acutely unwell; and other staff members trying to persuade the delegate to go elsewhere instead of the post-take ward round such as clinic.^2^ As the junior doctor, I was part of the simulation, and was directed to be disruptive to the ward round to see how the delegate would respond. Of course, this added an extra element of pressure to the simulation but a pressure that does reflect the realities of working in a team.

Ward rounds are one of the most important aspects of a junior doctor’s role within the multidisciplinary team. During medical school, students take part in these regularly as part of their placement. However, this did not stop me from feeling apprehensive when as a junior doctor, I experienced my first surgical ward round. As seen in previous studies, final year medical students are often not confident in actively taking part in ward rounds^3,^^4^ hence my apprehension towards this part of the job is unlikely to be unique as a newly qualified doctor. By helping at the urology boot camp, I was hopeful that it would help me become more confident and find ways to be more successful in this part of my role.

The scenarios were common urological problems that my team would often face, and the many interruptions are frequent occurrences that have to be dealt with by myself and my seniors. Observing my seniors manage the patients and distractions during the simulation, often in very different but effective ways, helped me to consider how I would act under the same stressors during a busy ward round. I was able to highlight actions I thought were done well and responses I felt could have been done differently. I even had the chance to feed this back during the debrief sessions after each delegate had been observed.

A very positive part of the day for me was realising how important we are as junior doctors in ensuring a smooth and effective ward round. When I was playing a more disruptive and unhelpful junior doctor it led to the lack of cohesiveness within the team. In particular it created a much more difficult time for the urology trainee trying to manage the patients as well as the distractions. During the times that I was more helpful and take initiative to contribute more, the trainee would often give me feedback that it was a much more positive experience and they felt I eased a lot of the pressure they were feeling during the simulation. This contrast highlighted the importance of my actions during the simulations.

The reason I found this so positive is that as the most junior member of the team it was easy to forget that you can be an asset and that people still value your opinion and help, despite being much less experienced than they are. Through the usual busy working day, people are often too busy to give feedback or thank you for your help. However, taking part in the boot camp reminded me that my role is valuable and can help to ease the pressures that my seniors face. This indirectly contributes to improved patient care. Being part of this simulation helped me consider how training for final year medical students leading up to the first day on the ward could be improved. At medical school I did take part in a number of simulations, however, they mainly focused on emergency situations focusing on the A to E approach to patient management.

Placements are the best opportunity to take part in a ward round, but as the surgical round is so quick I think medical students shy away from taking an active role such as writing in notes as they do not want to slow the ward round down. The main issue here is that although observing a ward round is useful, more active participation, a hands-on approach, is the best way to learn and contribute.

More training for final year medical students and new doctors is required to make them better prepared for ward rounds.^3^^-^^5^ There is lack of consensus regarding how best to prepare doctors transitioning from medical school to the clinical environment^5^ and the approach taken in the urology boot camp is one I think could be adopted in training. The simulated ward round highlighted the need for 1^st^ year junior doctors to become more proficient in being part of a surgical ward round.
